# Label-free proteomic analysis of serum exosomes from paroxysmal atrial fibrillation patients

**DOI:** 10.1186/s12014-020-09304-8

**Published:** 2021-01-06

**Authors:** Hanwen Ni, Wenqi Pan, Qi Jin, Yucai Xie, Ning Zhang, Kang Chen, Tianyou Lin, Changjian Lin, Yun Xie, Jiemin Wu, Peihua Ni, Liqun Wu

**Affiliations:** 1 Department of Cardiology, Ruijin Hospital, Shanghai Jiao Tong University School of Medicine, 197# Ruijin Rd, Huangpu District, Shanghai, 200025 China; 2 Faculty of Medical Laboratory Science, Ruijin Hospital, Shanghai Jiao Tong University School of Medicine, 197# Ruijin Rd, Huangpu District, Shanghai, 200025 China

**Keywords:** Atrial fibrillation (AF), Serum exosomes, Proteomic analysis

## Abstract

**Background:**

Atrial fibrillation (AF) is the most common cardiac heterogeneous rhythm disorder. It represents a major cause of mortality and morbidity, mainly related to embolic events and heart failure. Mechanisms of AF are complex and remain incompletely understood. Recent evidence suggests exosomes are membrane-coated objects released by many cell-types. Their presence in body fluids and the variable surface composition and content render them attractive as a mechanism for potential biomarkers. However, the content of serum exosomes of AF patients has not been fully delineated.

**Methods:**

In this work, the serum exosomes from AF patients and healthy donors were used to compare changes in the exosome protein content. Exosomes were isolated from serum of AF patients and healthy donors and their purity was confirmed by Western blotting assays and transmission electron microscopy (TEM). Label-free LC–MS/MS quantitative proteomic analysis was applied to analyze protein content of serum exosomes.

**Results:**

A total of 440 exosomal protein groups were identified, differentially expressed proteins were filtrated with fold change ≥ 2.0 (AF/controls protein abundance ratio ≥ 2 or ≤ 0.5) and p value less than 0.05 (p < 0.05), significantly changed in abundance group contains 39 elevated proteins and 18 reduced proteins, while consistent presence/absence expression profile group contains 40 elevated proteins and 75 reduced proteins. Bioinformatic analysis of differential exosomal proteins confirmed the significant enrichment of components involved in the anticoagulation, complement system and protein folding. Parallel-Reaction Monitoring Relative Quantitative Analysis (PRM) further suggested that AF related to complement system and protein folding.

**Conclusions:**

These results revealed the composition and potential function of AF serum exosomes, thus providing a new perspective on the complement system and protein folding to AF.

## Background

Atrial fibrillation (AF) is the most common chronic arrhythmia leading to adverse prognosis and having a significant impact on healthcare costs [1]. According to previous studies, AF was associated with a 1.5- to 1.9-fold mortality risk [2]. A worldwide epidemiology study also suggested the progressive increase in overall burden, incidence, prevalence, and AF-associated mortality [3].

Atrial fibrillation occurs when structural or electrophysiological abnormalities alter atrial tissue [4, 5]. These abnormalities are caused by diverse pathophysiological mechanisms, such that AF represents a final common phenotype for multiple disease pathways and mechanisms that are incompletely understood [1]. Several hypotheses have been proposed to explain the electrophysiological mechanisms that initiate and maintain AF. The multiple mechanisms likely coexist in an individual patient [6].

While the electrophysiological mechanism of AF remains controversial, its underlying biochemical mechanism was also not clearly explained. Several studies examined biomarkers in AF over the past decade, Troponin, B-type natriuretic peptide, D-dimer to name a few were potential indicators in AF stroke risk assessment evaluation [7]. However, few of the proposed markers revealed the possible pathophysiological genesis. Recent research found that exosomes were closely related to cardiovascular disease. The content of serum exosomes changes based on the type of cellular stress [8] and exosomes possibly played a pathologic role in the progression of cardiovascular disease [9].

Exosomes are nanosized membrane-derived vesicles (50–100 nm in diameter) secreted by a number of healthy and diseased cell types. Exosomes contain functional biomolecules (including proteins, RNA, DNA and lipids) that can be transferred to recipient cells with preservation of their function [10]. Characterization of exosomal cargo can provide clues to exosome biogenesis, targeting, and cellular effects and may be a source of biomarkers for disease diagnosis, prognosis and response to treatment. Both protein and RNA products have been used for these purposes. With recent improvements in proteomics technologies, both qualitative and quantitative characterization of exosomal proteins is possible. Modern molecular medicine is rapidly moving beyond functional genomics to proteomics [11]. The profile of proteins which are packaged into the serum exosomes may yield a molecular signature that is informative about physiological status and disease conditions induced by AF. The aim of this study was to demonstrate that the proteomics of serum exosome in AF and the better understood of exosomes in molecular mechanisms of AF.

## Materials and methods

### General experiment design

To investigate differences in the protein content of serum exosomes in AF patients and healthy donors. Exosomes were isolated from serum of 15 AF patients and 15 healthy donors and their purity was confirmed by Western blotting assays and TEM. The serum exosomes of 15 patients were randomly divided into three groups. The serum exosomes of 15 healthy donors were also randomly divided into three groups as a control specimen. Label-free LC–MS/MS Quantitative Proteomic Analysis was applied to analyze protein content of serum exosomes. Data were analyzed using Gene Ontology (GO) and Protein interaction network (PPI). Parallel-Reaction Monitoring (PRM) relative quantitative analysis assays were performed to confirm the mass spectrometry results of AF and control groups.

### Patients and serum samples

The serum samples were obtained from 15 healthy donors and 15 hospitalized paroxysmal AF patients. All patients experienced fatigue, palpitations, dyspnea, hypotension or syncope. All patients were diagnosed as atrial fibrillation by electrocardiogram (ECG). The ECG diagnostic criteria were (1) irregular R–R intervals, (2) absence of distinct repeating P Waves, and (3) irregular atrial activity [1]. AF of all patients recur with variable frequency but terminated within 7 days, either spontaneously or with intervention. All patients were refractory to at least one class I or III antiarrhythmic medication, undergoing new oral anticoagulant therapy and ready to receive AF catheter ablation. All samples were obtained from Ruijin Hospital Affiliated to Shanghai Jiao Tong University Medical School. The samples were obtained from the patients with informed consent and with approval of the institutional ethics committee. All paroxysmal atrial fibrillation in this study was defined according to the 2014 AHA/ACC/HRS Guideline for the Management of Patients with Atrial Fibrillation. The patient information is in Table 1 and the details can be found in Additional file 1: Table S1.

**Table 1:** Patient and control group information

	Patient	Normal	*p*
Age (years)	52.53 ± 7.19	43.47 ± 6.74	0.00
Women (%)	47	53	0.93
Hypertension (%)	53	26.67	0.15
Systolic BP (mm Hg)	128.4 ± 17.55	119 ± 14.94	0.12
Diastolic BP (mm Hg)	80.27 ± 10.76	66 ± 5.4	0.00
BMI (kg/m^2^)	25.09 ± 3.61	24.12 ± 1.37	0.34
NOAC (%)	93	0	N/A
Duration of disease (months)	37.7	0	N/A
Cholesterol (mmol/L)	4.05 ± 0.64	1.03 ± 0.33	0.00
HDL-C (mmol/L)	1.15 ± 0.24	1.48 ± 0.24	0.00
LDL-C (mmol/L)	2.4 ± 0.58	2.88 ± 0.5	0.02
Average left atrial dimension (mm)	39.33 ± 4.08	32.53 ± 2.67	0.00

### Exosome isolation from human serum

After centrifuged at 500*g* for 5 min of blood, serum placed in − 80 °C. Serum specimens were thawed and centrifuged at 2000*g* for 10 min at 4 °C and then at 12,000*g* for 30 min at 4 °C.

Clarified serum was passed through 0.22 μm-pore Millipore filter and used for exosome isolation by SEC performed using 1.5 ~ 12 cm mini columns (Bio-Rad, Hercules, CA, USA; Econo-Pac columns) packed with Sepharose 2B (Sigma-Aldrich, St. Louis, MO, USA). The column bed volume is 10 ml. Prior to applying clarified serum, the column was washed with phosphate-buffered saline (PBS) 20 ml, and a porous frit was placed at the top of the gel to prevent its disturbance during subsequent elution with PBS. Clarified serum 1.0 ml was loaded onto the column and five 1 ml fractions corresponding to the void volume peak were collected. Fractions No. 3, 4 and 5 were tested for protein measurements, western blot, proteomic analysis, morphology by transmission electron microscopy (TEM) and proteomic analysis [12].

### Western blots

In preparation for western blotting, the fractions were concentrated using 300,000 MWCO VivaSpin 500 Centrifugal Concentrators (Sartorius Corp, New York, NY, USA) by centrifugation at 5000*g* for 10 min, depending on the content.

Protein concentrations in isolated exosome fractions were measured using a BCA protein assay kit (Pierce Biotechnology, Rockford, IL, USA) according to the manufacturer’s instructions.

Isolated exosomes were tested for the exosomal markers, CD9, CD63 (Santa Cruz, Dallas, TX, USA) using western blots. Briefly, exosomes (10 mg protein) were separated on 12% SDS-PAGE gels and transferred onto the polyvinylidene fluoride (PVDF) membrane (Millipore, Billerica, MA, USA) for western blot analysis. Membranes were incubated overnight at 48 °C with antibodies specific for the designated antigens and purchased from Santa Cruz, Dallas, TX, USA: CD63 (1:200, sc-5275), CD9 (1:200, sc-13118); from Proteintech, Chicago, IL, USA:β-actin (1:2000, SA00001-1). Next, Anti-mouse IgG, HRP-linked Antibody (1:2000, Cell Signaling, Danvers, MA, USA:) was added for 1 h at room temperature (RT), and blots were developed with ECL detection reagents (GE Healthcare Biosciences). Band intensities on exposed films were quantified using Image J software (NIH, USA).

### Transmission electron microscopy (TEM)

TEM of isolated exosomes was performed at the Center for Biological Imaging at the School of Medical, Shanghai Jiao Tong University. Freshly isolated exosomes were put on a copper grid for 3 min at room temperature, coated with 2% phosphotungstic acid (PTA), negatively stained at room temperature for 3 min, filter paper to absorb the excess dye solution, then the stained copper grid with exosome was parched by the lamp for about 1 min. HITACHI H7650 TEM was used for imaging.

### Label-free quantitative proteomic analysis

#### Sample preparation

The serum exosomes of 15 patients were randomly divided into three groups. The serum exosomes of 5 patients in each group were balanced mixed. As a test sample, the serum exosomes of 5 patients in each group were detected in the same three groups. The serum exosomes of 15 normal persons were also selected and randomly divided into three groups. The serum exosomes of 5 patients in each group were balanced mixed as a control specimen, and the control samples were detected with the same three groups.

#### MS sample preparation

##### SDT lysis [13]

SDT buffer was added to AF serum exosomes. The lysate was sonicated and then boiled for 15 min. After centrifuged at 14,000*g* for 40 min, the supernatant was quantified with the BCA Protein Assay Kit (Bio-Rad, USA). The sample was stored at − 80 °C.

##### SDS-PAGE separation

20 µg of proteins for each sample were mixed with 5X loading buffer respectively and boiled for 5 min. The proteins were separated on 12.5% SDS-PAGE gel (constant current 14 mA, 90 min). Protein bands were visualized by Coomassie Blue R-250 staining.

##### Filter-aided sample preparation (FASP digestion) [13]

200 μg of proteins for each sample were incorporated into 30 μl SDT buffer (4% SDS, 100 mM DTT, 150 mM Tris–HCl pH 8.0). The detergent, DTT and other low-molecular-weight components were removed using UA buffer (8 M Urea, 150 mM Tris–HCl pH 8.0) by repeated ultrafiltration (Microcon units, 10 kD). Then 100 μl iodoacetamide (100 mM IAA in UA buffer) was added to block reduced cysteine residues and the samples were incubated for 30 min in darkness. The filters were washed with 100 μl UA buffer three times and then 100 μl 25 mM NH_4_HCO_3_ buffer twice. Finally, the protein suspensions were digested with 4 μg trypsin (Promega) in 40 μl 25 mM NH_4_HCO_3_ buffer overnight at 37 °C, and the resulting peptides were collected as a filtrate. The peptides of each sample were desalted on C18 Cartridges (Empore™ SPE Cartridges C18 (standard density), bed I.D. 7 mm, volume 3 ml, Sigma), concentrated by vacuum centrifugation and reconstituted in 40 µl of 0.1% (v/v) formic acid. The peptide content was estimated by UV light spectral density at 280 nm using an extinctions coefficient of 1.1 of 0.1% (g/l) solution that was calculated on the basis of the frequency of tryptophan and tyrosine in vertebrate proteins.

##### Mass spectrometry

HPLC: Each fraction was injected for nano LC–MS/MS analysis. The peptide mixture was loaded onto a reverse phase trap column (Thermo Scientific Acclaim PepMap100, 100 μm*2 cm, nanoViper C18) connected to the C18-reversed phase analytical column (Thermo Scientific Easy Column, 10 cm long, 75 μm inner diameter, 3 μm resin) in buffer A (0.1% Formic acid) and separated with a linear gradient of buffer B (84% acetonitrile and 0.1% Formic acid) at a flow rate of 300 μl/min controlled by IntelliFlow technology. The linear gradient was determined by 2 h gradient: 0–55% buffer B for 110 min, 55–100% buffer B for 5 min, hold in 100% buffer B for 5 min.

LC–MS/MS analysis: LC–MS/MS analysis was performed on a Q Exactive mass spectrometer (Thermo Scientific) that was coupled to Easy nLC (Proxeon Biosystems, now Thermo Fisher Scientific) for 240 min. The mass spectrometer was operated in positive ion mode. MS data was acquired using a data-dependent top 10 method dynamically choosing the most abundant precursor ions from the survey scan (300–1800 m/z) for HCD fragmentation. Automatic gain control (AGC) target was set to 3e6, and maximum inject time to 10 ms. Dynamic exclusion duration was 40.0 s. Survey scans were acquired at a resolution of 70,000 at m/z 200 and resolution for HCD spectra was set to 17,500 at m/z 200, and isolation width was 2 m/z. Normalized collision energy was 30 eV and the underfill ratio, which specifies the minimum percentage of the target value likely to be reached at maximum fill time, was defined as 0.1%. The instrument was run with peptide recognition mode enabled [14].

##### Data analysis

The MS data were analyzed using MaxQuant software version 1.3.0.5 (Max Planck Institute of Biochemistry in Martinsried, Germany) [15]. The following parameters were set, Enzyme = Trypsin, Max Missed Cleavages = 2, Main search = 6 ppm, First search = 20 ppm, MS/MS Tolerance = 20 ppm, Peptide FDR ≤ 0.01, Protein FDR ≤ 0.01, Time window (match between runs) = 2 min, Protein Quantification = Razor and unique peptides were used for protein quantification. LFQ [14] = True, LFQ min. ratio count = 1.

### Differentially expressed protein analysis

The differentially expressed proteins in AF serum exosome samples compared to normal control samples were identified with |Abundance Ratio ≥ 2 or ≤ 0.5, p value < 0.05 was considered significant. Hierarchical clustering of the expression of the differentially expressed proteins was performed by heatmap. The data can be found in Additional file 2: Table S2.

### Bioinformatic analysis

The sequence data of the selected differentially expressed proteins were retrieved in batches from the UniProtKB database (Release 2013_12) in FASTA format. The retrieved sequences were locally searched against the SwissProt database (human) using the NCBI BLAST + client software (ncbiblast-2.2.28+-win32.exe) to find homolog sequences so that the identified sequences can be functionally annotated. The top 10 blast hits with an E-value less than 1e−3 for each query sequence were retrieved and loaded into Blast2GO [16] (Version 2.7.0) for gene ontology (GO) [17] mapping and annotation. An annotation configuration with an E-value filter of 1e−6, default gradual enzyme codes (EC) weights, a GO weight of 5, and an annotation cutoff of 90 were chosen. Unannotated sequences were then reannotated with more permissive parameters. Sequences without BLAST hits and unannotated sequences were then selected for InterProScan [18] against EBI databases to retrieve functional annotations of protein motifs and merge the InterProScan GO terms to the annotation set. The protein–protein interaction network was generated by STRING 10 (http://string-db.org/) [19]. The data can be found in Additional file 3: Table S3.

### PRM MS analysis

Target analysis by Parallel reaction monitoring (PRM). The targeted quantification and verification were carried out 19 target peptides of 9 target proteins were quantitatively analyzed by PRM. PRM analysis was performed by Q-Exactive HF mass spectrometer (Thermo Scientific Bremen, Germany) with 60 min. The MS acquisition mode was a combination of two scan events: a full scan and a time-scheduled scan. The full scan was taken at a resolution of 60,000 at m/z 200 with a scan mass range of 300 to 1800 m/z, a target (AGC) of 3e6 and maximum injection fill time is 200 ms. The scheduled scan was employed at a resolution of 30,000 at m/z 200, a target AGC of 3e6, and maximum injection fill time is 120 ms. Precursor ions were fragmented with normalized collision energy 27 [20].

Peptide digests were loaded onto a trap column (100 μm × 2 cm, Nanoviper) at a flow rate of 3 μl min^−1^. For PRM analysis, 2 μl of serum exosome peptides from each group were separated on an analytical column (75 μm × 50 cm, RSLC C18). Linear gradient ranging from 30% to 100% buffer B over 60 min was used. For each target protein, multiple peptides were monitored using a targeted inclusion list.

The raw data from target proteomics analysis were analyzed by software Skyline 3.5.0 [21]. Results were exported for additional data and statistical analysis (Additional file 4: Table S4). PRM data were further calculated in Table 3.

## Results

### Serum exosomes proteomic profiles of AF patient and normal show marked differences in protein expression

**Fig. 1:**
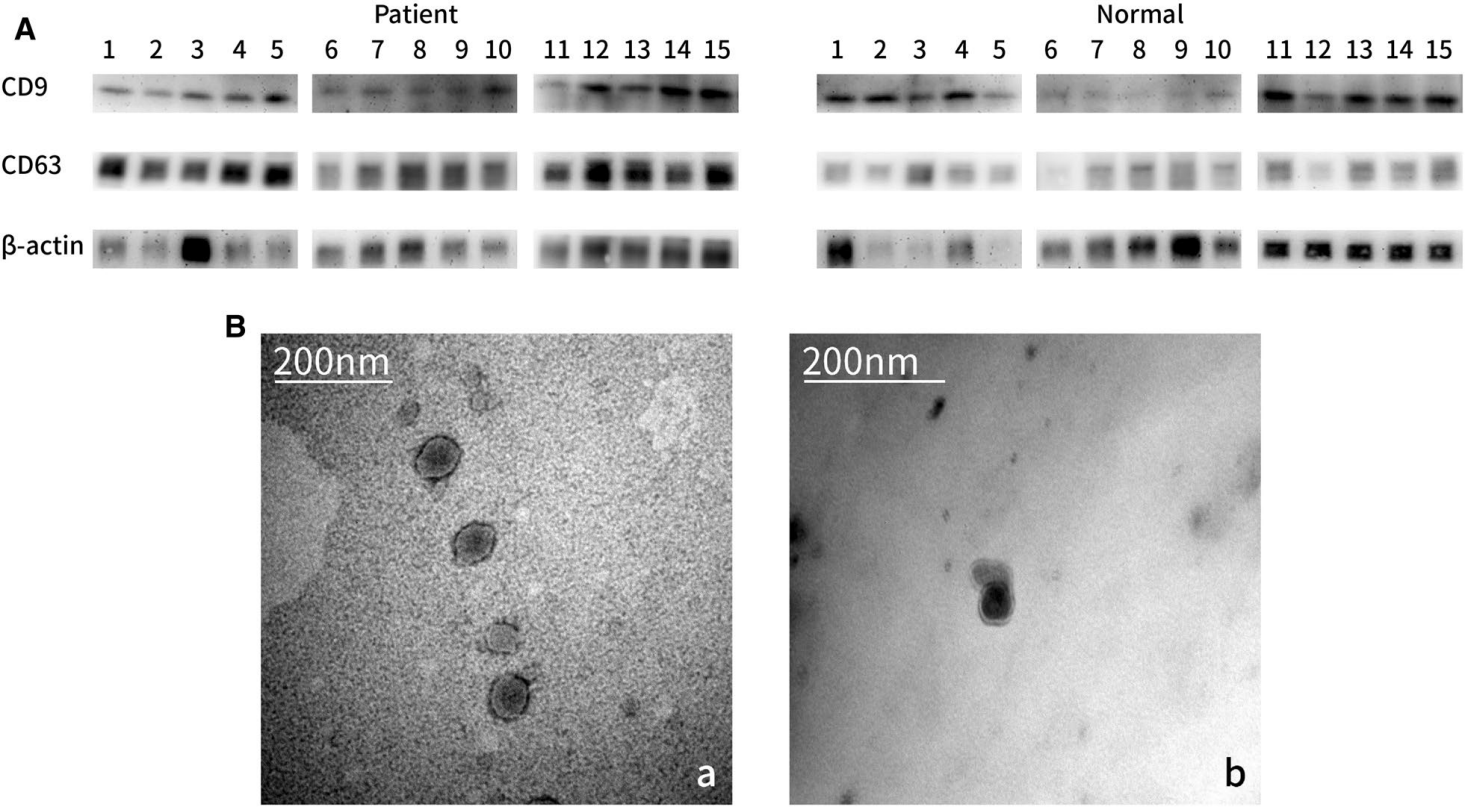
Molecular protein profiles of plasma exosomes from patients with AF and control groups. **A** Western blots were performed after loading 10 mg of exosomal protein/lane. Protein profiles are shown for exosomes of patients with AF and of controls. CD9, CD63 serves as an exosome marker. **B** TEM images of exosomes isolated from serum of patients with AF and controls. Exosomes appear as vesicles ranging in size from 30 to 100 nm. **a** exosomes of AF patients, **b** exosomes of healthy donors

**Fig. 2:**
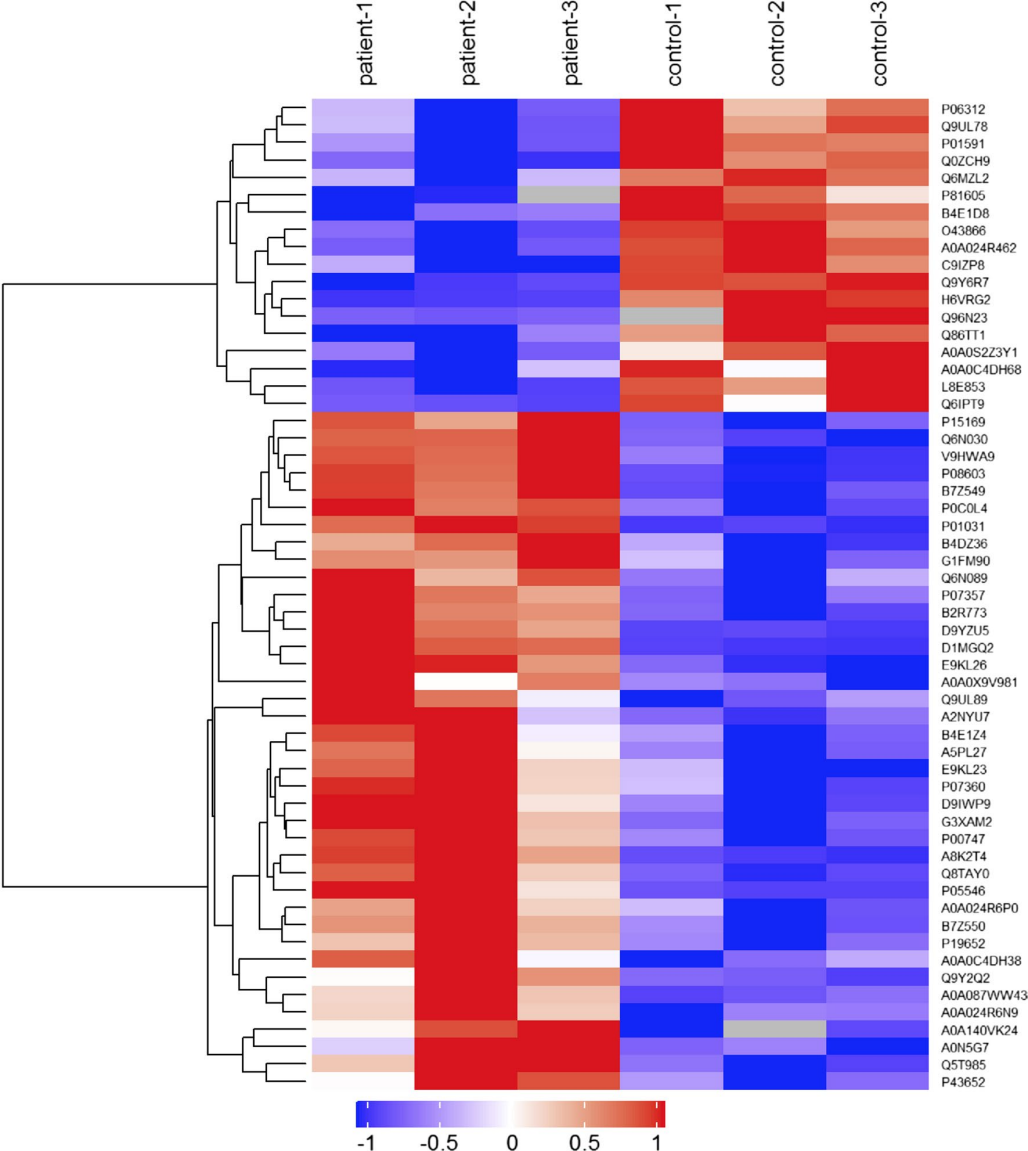
Differentially expressed protein profiles between AF serum exosome and controls. Total of 440 proteins are identified in patients, with 39 Proteins significantly increased, and 18 decreased in abundance, 40 consistent presence, 75 decreased by greater than two-fold and p < 0.05. Volcanic maps showed the significant changing of proteins between the patients and controls. The ratio of expression of these proteins in patients to controls are plotted against the p value. Selected proteins with a greater than two-fold change in expression are annotated. The red spots indicate proteins in significant changing. The black spots indicate proteins had no significant difference. Log2 expression values of the significantly differentially expressed proteins in different samples are displayed in the thermograph in different colors. Red and blue indicate up- or down regulation respectively, grey indicates no protein quantitative information

Serum exosomes from 15 AF patients and 15 normal volunteers as control groups were separated and verified exosomes markers CD9, CD63 by western blot. TEM also observed the exosomes (Fig. 1). Serum exosomes of AF patients and normal were grouped and done by label-free quantitative proteomic analysis. A total of 440 proteins were identified (Additional file 2: Table S2). Differentially expressed proteins were filtrated with fold change ≥ 2.0 (AF/controls protein abundance ratio ≥ 2 or ≤ 0.5) and *p* value less than 0.05 (*p *< 0.05). The accession numbers, gene names, score, unique peptides and relative ratios of the differentially expressed proteins are listed in Additional file 2: Table S2. Significantly changed in abundance group contains 39 elevated proteins and 18 reduced proteins, while consistent presence/absence expression profile group contains 40 elevated proteins and 75 reduced proteins. Hierarchical Cluster showed the differentially expressed proteins of serum exosomes of AF compared to the control groups (Fig. 2), original quantitative data for the heatmap can be found in Additional file 2: Table S2.

### Bioinformatic analysis of serum exosomes proteomic profiles of AF

**Fig. 3:**
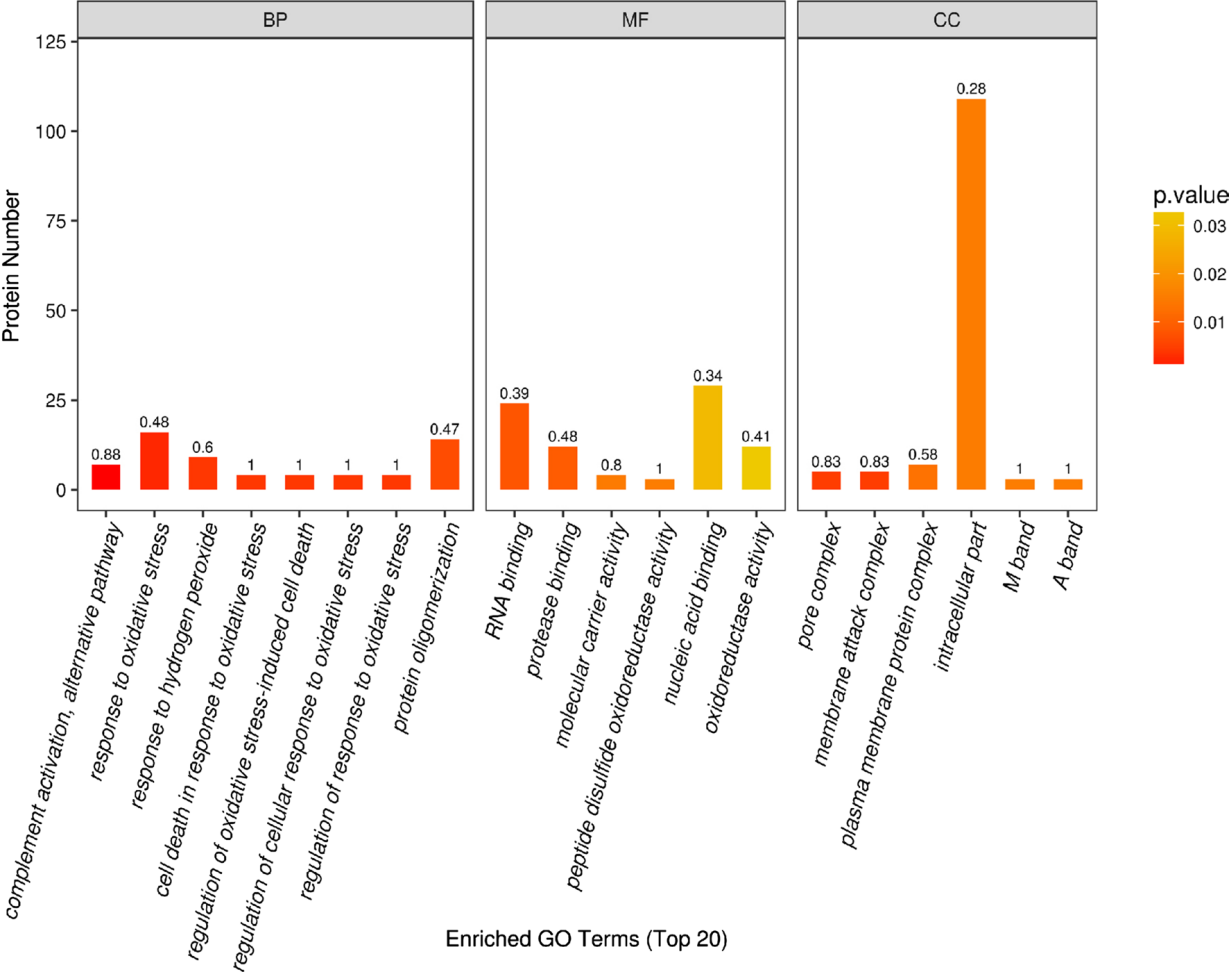
Gene Ontology (GO) analyses of the proteins in AF serum exosome and controls. GO function included three categories: biological processes (BP), cell components (CCs), and molecular function (MF) The abscissa in the graph represents the enriched GO functional classification, the ordinate represents the number of differentiated proteins under each functional classification; and the bar color represents the significance of enriched GO functional classification, i.e. based on Fisher exact test. (Fisher’s Exact Test) calculates *p* value. The color gradient represents the size of *p* value. The color gradient changes from orange to red, and the closer the red represents *p* value, the higher the significance level of GO functional category enrichment. The upper label on bar chart shows richFator <= 1, and the enrichment factor represents the number of differentially expressed proteins annotated to a GO functional category, proportion of all identified proteins released into the GO functional category

To study the changes of differentially expressed proteins of serum exosomes of AF, it is important to understand functions, cellular localization and biological processes of each protein. Therefore, it is necessary to systematic summarize and analyze the proteins and their functions. We used Blast2Go (https://www.blast2go.com/) to perform Gene Ontology (GO) function annotation and Fisher’s Exact Test for GO functional enrichment analysis. GO functional analysis included three categories: biological processes (BP), molecular function (MF), and cell components (CCs) (Fig. 3). In biological processes (BP), these processes significantly changed in complement activation, alternative pathway; response to oxidative stress; response to hydrogen peroxide; protein oligomerization. In molecular function (MF), RNA binding, nucleic acid binding and oxidoreductase activity are the major functional classes. In cell components (CCs), the changes of isolocalized proteins enriched in intracellular part, plasma membrane protein, pore complex and membrane attack complex. GO functional classification is calculated based on Fisher’s Exact Test. The color gradient represents the size of *p* value. The color gradient changes from orange to red. The closer to red, the smaller the *p* value and the higher the saliency level of corresponding GO functional categories. The smaller the *p* value of GO enrichment results (*p *< 0.05), the more significant the corresponding GO functional classification is statistically enriched. The number of differentially expressed proteins related to GO functional classification reflects to some extent the degree of influence of biological treatment on each classification in experimental design. Therefore, the *p* value and the number of differentially expressed proteins can be combined to select more interesting biological functions. The differentially expressed proteins that significantly affect these functions will be verified by subsequent biological experiments or mechanism studies. Based on this research, the differentially expressed proteins that affect these functions will focus on the processes of complement activation, response to oxidative stress, protein oligomerization.

**Fig. 4:**
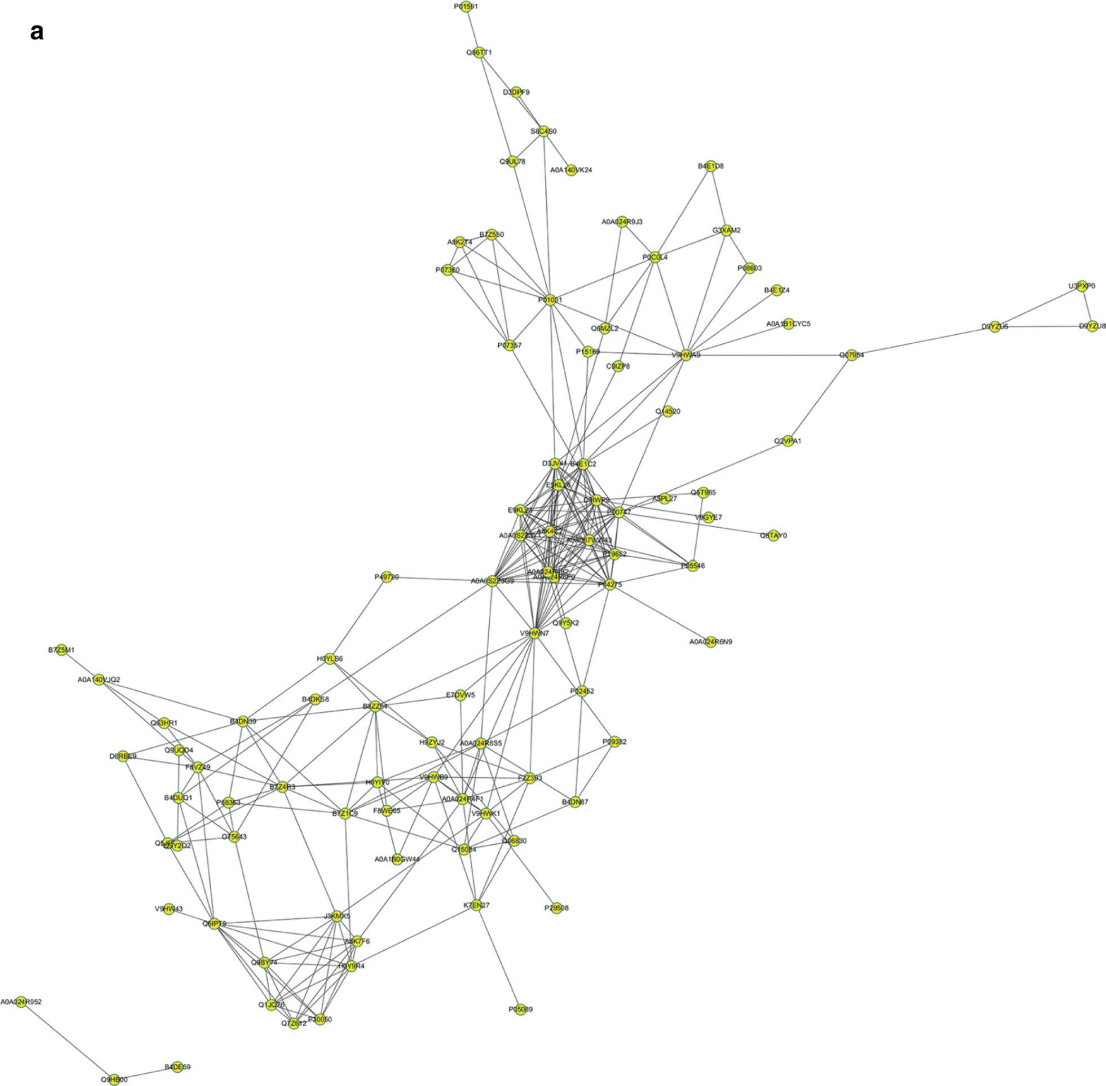
Three relatively concentrated nets were obtained by PPI analysis. These changes were manifested in blood anticoagulation, changes of immune system, and changes related to protein folding (**a**)

To further understand protein–protein interactions in the differentially expressed proteins, IntAct Uniprot and MINT database were queried to determine the interaction between target proteins and other proteins directly acting with them. CytoScape software was used to generate interaction network. Three relatively concentrated nets were obtained by protein–protein interactions (PPI) analysis (Fig. 4). The proteins were enriched in blood anticoagulation, immune system, and protein folding. The proteins involved in immune system, and protein folding consistent with the result of GO analysis (Table 2). Indeed, the accurate regulation of cardiac proteostasis may be impaired by several stress conditions, including oxidative stress, causing an accumulation of damaged and misfolded proteins that exceed the cellular degradation ability. Unfolded proteins can thus aggregate in toxic oligomers and finally in bigger insoluble aggregates disrupting cardiomyocyte structure and function and leading to cardiomyopathy [22]. Therefore, combined with the results of GO and PPI, we will focus on the protein folding that also involve in oxidative stress. In addition to immune system, particularly complement system will be the other target.

**Table 2:** Proteins selected from PPI analysis

Protein group	Protein name	Function
A8K2T4	C7, Complement component 7	Constituent of the membrane attack complex (MAC) that plays a key role in the innate and adaptive immune response by forming pores in the plasma membrane of target cells. C7 serves as a membrane anchor
G3XAM2	CFI, Complement factor I	Responsible for cleaving the alpha-chains of C4b and C3b in the presence of the cofactors C4-binding protein and factor H respectively
B7Z550	C8B, Complement component 8, beta polypeptide	Constituent of the membrane attack complex (MAC) that plays a key role in the innate and adaptive immune response by forming pores in the plasma membrane of target cells
P07357	C8A, Complement component 8, alpha polypeptide	Constituent of the membrane attack complex (MAC) that plays a key role in the innate and adaptive immune response by forming pores in the plasma membrane of target cells. C8A inserts into the target membrane, but does not form pores by itself
P07360	C8G, Complement component 8, gamma polypeptide	C8 is a constituent of the membrane attack complex. C8 binds to the C5B-7 complex, forming the C5B-8 complex. C5-B8 binds C9 and acts as a catalyst in the polymerization of C9. The gamma subunit seems to be able to bind retinol
P08603	CFH, Complement factor H	Factor H functions as a cofactor in the inactivation of C3b by factor I and increases the rate of dissociation of the C3bBb complex (C3 convertase) and the (C3b) NBB complex (C5 convertase) in the alternative complement pathway
Q6IPT9 EEF1A1	Eukaryotic translation elongation factor 1 alpha 1	This protein promotes the GTP-dependent binding of aminoacyl-tRNA to the A-site of ribosomes during protein biosynthesis. With PARP1 and TXK, forms a complex that acts as a T helper 1 (Th1) cell-specific transcription factor and binds the promoter of IFN-gamma to directly regulate its transcription, and is thus involved importantly in Th1 cytokine production
Q15084	PDIA6, Protein disulfide isomerase family A, member 6	May function as a chaperone that inhibits aggregation of misfolded proteins. Plays a role in platelet aggregation and activation by agonists such as convulxin, collagen and thrombin
F8WE65	PPIA, Peptidylprolyl isomerase A (cyclophilin A)	PPIases accelerate the folding of proteins. It catalyzes the *cis*–*trans* isomerization of proline imidic peptide bonds in oligopeptides

### Studied and identified the protein expression by PRM

GO and PPI analysis result suggests that immune system and protein folding, especially in complement system has significantly altered in the AF. We tried to verify the association between immune system, protein folding and AF by PRM relative quantitative Analysis. From the differentially expressed proteins (S2), we selected 9 proteins were quantitatively analyzed by LC-PRM/MS. In 9 proteins, complement 7 (C7), complement 8 (C8), complement 5 (C5), complement factor I, complement factor B, complement factor H are relevant to immune system. The other three proteins with the functions in protein synthesis and protein folding were selected: elongation factor 1-alpha, protein disulfide-isomerase and Peptidyl–prolyl *cis*–*trans* isomerase (Table 3).

**Table 3:** Proteins selected in immune system and protein folding monitored by PRM/MS

Protein group	Pathway	Signature peptide(s)	Normalized peak area						Relative abundance of protein		Ratio	TTEST
			Patient 1	Patient 2	Patient 3	Control 1	Control 2	Control 3	Patient average	Control average	Patient/Control	
A8K2T4	Prion diseases	LIDQYGTHYLQSGSLGGEYR QNDFNSVEEK SSGWHFVVK	0.2679	0.3582	0.3529	0.1314	0.0987	0.0911	0.3263	0.1071	3.05	0.00231
G3XAM2	*Staphylococcus aureus* infection Complement and coagulation cascades	HGNTDSEGIVEVK, IVIEYVDR	1.6821	1.4129	1.3069	0.7351	0.6307	0.7247	1.4673	0.6968	2.11	0.0027
B4E1Z4	Complement and coagulation cascades, *Staphylococcus aureus* infection	EELLPAQDIK, QLNEINYEDHK	16.2201	12.5770	9.5189	7.9080	2.6827	5.3475	12.7720	5.3127	2.40	0.0385
B7Z550	Prion diseases Complement and coagulation cascades, Systemic lupus erythematosus, Amoebiasis	EYESYSDFER, LPLEYSYGEYR, QALEEFQK, SGFSFGFK	1.6165	1.6726	1.1954	0.8485	0.3616	0.5914	1.4949	0.6005	2.49	0.0122
P08603	Complement and coagulation cascades, *Staphylococcus aureus* infection	GEWVALNPLR, IVSSAMEPDR, NGFYPATR	30.7818	20.2144	22.8060	16.6629	12.3889	13.2042	24.6007	14.0853	1.75	0.0377
P01031	Systemic lupus erythematosus, Pertussis, Herpes simplex infection, Prion diseases, Complement and coagulation cascades, *Staphylococcus aureus* infection	DSEITFIK, IDTQDIEASHYR	3.2947	2.6653	2.4174	1.2760	0.6331	0.8486	2.7925	0.9192	3.04	0.0044
Q6IPT9	RNA transport, Legionellosis	IGGIGTVPVGR	0.4415	0.1840	0.2476	0.6172	0.3933	0.4184	0.2910	0.4763	0.61	0.1522
A0A024R8S5	Protein processing in endoplasmic reticulum	EADDIVNWLK	0.0297	0.0277	0.0265	0.0220	0.0348	0.0441	0.0280	0.0336	0.83	0.4315
F8WE65	Necroptosis	VSFELFADK	0.0246	0.0350	0.0421	0.0387	0.0383	0.0469	0.0339	0.0413	0.82	0.2716

The 19 peptide segments of 9 target proteins in 6 sample groups were quantitatively analyzed by LC-PRM/MS and analyzed by Skyline (https://skyline.ms/). The quantitative information of target peptide segments was found, the details are shown in Additional file 4: Table S4. Quantitative information was normalized by isotope rescaled peptide fragments, and then the target peptide fragments and target proteins were quantitatively analyzed. The results of differential multiples and TTEST test showed that there were some differences in the expression of 9 target proteins under two different conditions, AF and control groups (Table 3).

We performed PRM assays to confirm the mass spectrometry results of AF and control groups. The expression levels of these 9 proteins showed a consistent trend with the mass spectrometry results. We found that C7, C8, C5, complement factor I, complement factor B, complement factor H, protein expression levels were upregulated in AF patients, and elongation factor 1-alpha, protein disulfide-isomerase and Peptidyl–prolyl *cis*–*trans* isomerase were downregulated in AF patients. The effects of 9 proteins are on complement system and protein folding in AF patients.

## Discussion

This is the first proteomic analysis in serum exosomes of AF patients. The proteomic analysis is based on pooled samples. In our study, comparison of serum exosomes from AF patients with healthy volunteers by label-free LC–MS/MS quantitative proteomic yielded many differentially expressed proteins, revealing obvious differences in complement activation and protein folding.

AF is the most common chronic arrhythmia leading to adverse prognosis and having a significant impact on healthcare costs [1]. Ageing, oxidative stress and inflammation are the key risk factors of atrial fibrillation. With the development of age-related cardiovascular disease, increased production of reactive oxygen species and systemic inflammation promote cardiac structural and electrophysiologic remodeling. AF is a heterogeneous heart rhythm disorder related to a wide spectrum of etiologies and has broad clinical presentations. Despite extensive research, the mechanisms underlying AF remain incompletely understood.

The mechanisms underlying AF are classically described as mechanisms responsible for its initiation and mechanisms responsible for its perpetuation [23]. In this study, a multi-stage discovery-verification proteomic strategy was used to develop potential AF-related biomarkers from patients’ blood samples. All patients are symptomatic paroxysmal atrial fibrillation, refractory to at least 1 class I or III antiarrhythmic medication, undergoing new oral anticoagulant therapy and ready to accept AF catheter ablation.

Exosomes are released from multiple cell types. Exosomes contain protein and RNA species and have been exploited as a novel reservoir for disease biomarker discovery. The molecular content, including proteins, of exosomes are heavily dependent on the tissue/cell-type derived from. Exosomes from diverse origins contain a conserved set of proteins as well as a subset of cell type/tissue specific proteins. Many diseases alter the proteins and RNAs of exosomes in bodily fluids including plasma, serum, urine of patients. This is now being investigated as a potential source of biomarkers. Studies in cardiovascular diseases are now focused on the contents of these vesicles, either microRNAs or proteomic analysis of cardiac exosomes under normal and disease conditions. Exosomes were recently found involved in the pathologic process in the progression of cardiovascular disease. The content of exosomes also changes based on the type of cellular stress [8]. But proteomic analysis of bodily fluids in AF is still unknown. In our study, we focus on the serum exosomes proteomic analysis in AF patients, which might lead to further understanding of the initiation, maintenance, progression and biomarkers of AF on molecular biology level.

Serum exosomes were obtained by exosome separation method and were identified by electron microscopy and Western Blot detection of CD63 and CD9. Serum exosomes of 15 patients with paroxysmal AF and 15 normal subjects were divided into 3 study groups and control groups, and the Label Free relative quantitative proteomics analysis was performed and identified a total of 440 proteins in serum exosomes. Differentially expressed proteins were obtained by standard screening of fold change ≥ 2 and *p* value < 0.05. Significantly changing in abundance group contains 39 elevated proteins and 18 reduced proteins. Consistent presence/absence expression profile group contains 40 elevated proteins and 75 reduced proteins. GO functional enrichment analysis and PPI network analysis found these pathways have significantly changed: complement activation and protein folding. 9 proteins involved in complement activation and protein folding were selected to perform PRM verification. Proteomics provide large quantities of data and many candidate proteins between a normal and disease condition. These candidates are normally subjected to use conventional techniques such as western blotting, ELISA and immunofluorescence to be validated. But there are problems that the antibodies and many novel proteins how to be detected. PRM provides not only an alternative in the lab but also high-grade quantification in the clinic. In this way multiple proteins (1–20) can be detected from one sample allowing the rapid identification of a panel of proteins and biomarkers. Novel biomarkers may not have established antibodies and reagents for conventional assays from patient samples. The PRM analysis result confirmed that patients with AF had a significant change in complement system and protein folding.

There are relatively few studies on atrial fibrillation and complement. Past studies suggested possible relationship between increased CRP level, complement activation and the increased risk of post-surgery arrhythmia. With increased inflammation, as defined by serum levels of CRP, C3 and C4, the risk of atrial fibrillation increase. But complement factors C3 and C4 separately failed to predict risk of atrial fibrillation, whereas a combination of inflammation sensitive proteins, including C3 and C4 [24, 25].

The complement system is a complex protein network of the innate immune system. It consists of soluble and membrane-bound proteins functioning in cascades of stepwise protease activation. Complement factors can be activated by three major pathways, the classical pathway, the lectin pathway and the alter-native pathway. Activation of any of the three pathways can lead to the cleavage of C3, and subsequent activation of C5, C6, C7, C8 and C9 of the terminal pathway. C1q and MBL are pattern recognition molecules of the classical and the lectin pathway, respectively; C1s, C1r, MBL-associated serine proteases (MASP), C2 and C4 further participate in classical and lectin pathway activation of C3. The alternative pathway activates C3 spontaneously in combination with Factor B (FB), Factor D (FD) and properdin. Furthermore, activation of C3 and C5 via extrinsic proteases of the coagulation, fibrinolysis and the kinin systems have nowadays been recognized as a fourth complement activation pathway [26, 27].

Inflammation is an important risk factor of atrial fibrillation. Complement mediated inflammation has been proved an important player in a variety of heart diseases [24, 28]. The role for the complement system is also in systemic inflammation. C3, C3a, C5a and C5b-9 were, independently of confounding factors, associated with systemic low-grade inflammation. The expression of C7, C8, C5, complement factor I, complement factor B, complement factor H were upregulated in AF patients may be accompanied by inflammation [29, 30]. Significant changes in complement activation are found in the serum exosome, because exosomes came from cells and tissues, which can better reflect the changes of cells and tissues under pathological conditions. Proteomics analysis in the serum exosome. The activation of complement system is accompanied by inflammation. AF is closely related to inflammation, meanwhile the change of complement system may be another reason for AF. The exact role of complement in AF will help to identify patients that might benefit from therapeutic complement intervention.

Atrial fibrillation (AF) is an age-related atrial tachyarrhythmia. A hallmark of aging means gradual derailment of proteostasis, including the homeostasis of protein synthesis, folding, assembly, trafficking, function, and degradation [31, 32]. The derailment of proteostasis during aging is an important factor for the development of age-related atrial fibrillation [32]. Protein homeostasis (proteostasis) is the maintenance of a functional equilibrium between protein synthesis, fidelity, folding, localization, modification, and degradation. The ability to maintain equilibrium in response to internal and external cues is essential for cellular and organismal health. Loss of proteostasis is a “hallmark” of aging [33].

Cellular protein synthesis consists of three distinct stages: initiation, elongation and termination, and all stages depend on translation factors. Eukaryotic elongation factor 1 alpha (eEF1A) delivers aminoacyl-tRNA to the ribosome and thereby plays a key role in protein synthesis [34]. Besides its essential role in the protein synthesis machinery, several non-canonical functions have also been described for eEF1A, such as regulation of the actin cytoskeleton and the promotion of viral replication. The functional significance of the extensive lysine methylations on eEF1A [35].

Protein disulfide isomerase (PDI) family are enzymes in productive protein folding accompanied by disulfide formation. PDIs have multiple functions in different states. PDIs are also a family of thioredoxin superfamily thiol oxidoreductase chaperones. PDIs have diverse functions in the endoplasmic reticulum [36]. PDIs play in thiol switches involving oxidation, reduction, isomerization and protein oligomerization. PDIs have the roles in oxidase activation and cell migration in vascular cells and macrophages. PDIs have peculiar redox/chaperone properties in redox signaling, which made them possible therapeutic targets [37].

The prolyl *cis*–*trans* isomerase (PIN1) is known to alter the structure of several proteins and stability of the proteins [38, 39]. PIN1-mediated isomerization alters the structure and activity of these proteins, regulating functions in cell metabolism, cell mobility, tumor development, oxidative stress and inflammation [40].

We demonstrated that altering of protein folding in the proteomics of serum exosome in AF patients. eEF1A, PDI and PIN1 play important role in protein synthesis and protein folding. The proteins expression decreased in serum exosomes in AF indicating the protein misfolded and unfolded. That also indicated the loss of proteostasis and aging. Misfolded or unfolded proteins have been found to play a role in arrhythmogenesis during human heart failure. Oxidative stress Reactive and oxygen species (ROS) are also the mechanism of AF [31]. ROS favor accumulation of misfolded proteins, in turn, further enhances oxidative stress. The expression of eEF1A, PDI and PIN1 decreased in serum exosomes in AF may break proteostasis and accelerate the oxidative stress and ROS. Blocking of misfolded or unfolded proteins may have an antiarrhythmic effect.

## Conclusions

Serum exosomes showed great promise for providing new insights into the mechanism of the initiation, maintenance, and progression of AF. The proteomics of serum exosomes indicated complement activation and altering of protein folding in AF patients. This is another sight in the mechanism of AF. With more understanding of serum exosomes, it might be helpful in early diagnostics of atrial fibrillation and, hopefully, monitoring the preoperative and postoperative rhythm control condition of catheter ablation.

## Supplementary information

**Additional file 1: Table S1.** Patient and control group information.

**Additional file 2: Table S2.** List of protein quantification and differential analysis.

**Additional file 3: Table S3.** PPI analysis.

**Additional file 4: Table S4.** Skyline data of target peptide PRM quantitative analysis.

## Data Availability

All data are stored in the form of an electronic database together and results from analysis in the form of a statistical software report.

## Abbreviations

AF: Atrial fibrillation
LC–MS/MS: Liquid chromatography-tandem mass spectrometry
GO: Gene Ontology
PPI: Protein–protein interaction
PRM: Parallel reaction monitoring

## References

[1] January CT, Wann LS, Alpert JS, Calkins H, Cigarroa JE, Cleveland JC, Conti JB, Ellinor PT, Ezekowitz MD, Field ME (2014). 2014 AHA/ACC/HRS guideline for the management of patients with atrial fibrillation: a report of the American College of Cardiology/American Heart Association Task Force on practice guidelines and the Heart Rhythm Society. Circulation.

[2] Benjamin EJ, Wolf PA, D’Agostino RB, Silbershatz H, Kannel WB, Levy D (1998). Impact of atrial fibrillation on the risk of death: the Framingham Heart Study. Circulation.

[3] Lane DA, Skjoth F, Lip GYH, Larsen TB, Kotecha D (2017). Temporal trends in incidence, prevalence, and mortality of atrial fibrillation in primary care. J Am Heart Assoc.

[4] Savelieva I, Kakouros N, Kourliouros A, Camm AJ (2011). Upstream therapies for management of atrial fibrillation: review of clinical evidence and implications for European Society of Cardiology guidelines. Part I: primary prevention. Europace..

[5] Wakili R, Voigt N, Kaab S, Dobrev D, Nattel S (2011). Recent advances in the molecular pathophysiology of atrial fibrillation. J Clin Investig.

[6] Calkins H, Kuck KH, Cappato R, Brugada J, Camm AJ, Chen SA, Crijns HJ, Damiano RJ, Davies DW, DiMarco J (2012). 2012 HRS/EHRA/ECAS Expert Consensus Statement on Catheter and Surgical Ablation of Atrial Fibrillation: recommendations for patient selection, procedural techniques, patient management and follow-up, definitions, endpoints, and research trial design. Europace.

[7] Hijazi Z, Oldgren J, Siegbahn A, Granger CB, Wallentin L (2013). Biomarkers in atrial fibrillation: a clinical review. Eur Heart J.

[8] Poe AJ, Knowlton AA (2017). Exosomes as agents of change in the cardiovascular system. J Mol Cell Cardiol.

[9] Malik ZA, Kott KS, Poe AJ, Kuo T, Chen L, Ferrara KW, Knowlton AA (2013). Cardiac myocyte exosomes: stability, HSP60, and proteomics. Am J Physiol Heart Circ Physiol.

[10] Colombo M, Raposo G, Thery C (2014). Biogenesis, secretion, and intercellular interactions of exosomes and other extracellular vesicles. Annu Rev Cell Dev Biol.

[11] Zhang P, Wang W, Wang X, Wang X, Song Y, Han Y, Zhang J, Zhao H (2013). Protein analysis of atrial fibrosis via label-free proteomics in chronic atrial fibrillation patients with mitral valve disease. PLoS ONE.

[12] Hong CS, Funk S, Muller L, Boyiadzis M, Whiteside TL (2016). Isolation of biologically active and morphologically intact exosomes from plasma of patients with cancer. J Extracell Vesicles.

[13] Wisniewski JR, Zougman A, Nagaraj N, Mann M (2009). Universal sample preparation method for proteome analysis. Nat Methods.

[14] Cox J, Hein MY, Luber CA, Paron I, Nagaraj N, Mann M (2014). Accurate proteome-wide label-free quantification by delayed normalization and maximal peptide ratio extraction, termed MaxLFQ. Mol Cell Proteomics.

[15] Cox J, Mann M (2008). MaxQuant enables high peptide identification rates, individualized p.p.b.-range mass accuracies and proteome-wide protein quantification. Nat Biotechnol.

[16] Gotz S, Garcia-Gomez JM, Terol J, Williams TD, Nagaraj SH, Nueda MJ, Robles M, Talon M, Dopazo J, Conesa A (2008). High-throughput functional annotation and data mining with the Blast2GO suite. Nucleic Acids Res.

[17] Ashburner M, Ball CA, Blake JA, Botstein D, Butler H, Cherry JM, Davis AP, Dolinski K, Dwight SS, Eppig JT (2000). Gene ontology: tool for the unification of biology. The Gene Ontology Consortium. Nat Genet.

[18] Quevillon E, Silventoinen V, Pillai S, Harte N, Mulder N, Apweiler R, Lopez R (2005). InterProScan: protein domains identifier. Nucleic Acids Res.

[19] Szklarczyk D, Franceschini A, Wyder S, Forslund K, Heller D, Huerta-Cepas J, Simonovic M, Roth A, Santos A, Tsafou KP (2015). STRING v10: protein-protein interaction networks, integrated over the tree of life. Nucleic Acids Res.

[20] Yan W, Luo J, Robinson M, Eng J, Aebersold R, Ranish J (2011). Index-ion triggered MS2 ion quantification: a novel proteomics approach for reproducible detection and quantification of targeted proteins in complex mixtures. Mol Cell Proteomics.

[21] MacLean B, Tomazela DM, Shulman N, Chambers M, Finney GL, Frewen B, Kern R, Tabb DL, Liebler DC, MacCoss MJ (2010). Skyline: an open source document editor for creating and analyzing targeted proteomics experiments. Bioinformatics.

[22] McLendon PM, Robbins J (2015). Proteotoxicity and cardiac dysfunction. Circ Res.

[23] Cheniti G, Vlachos K, Pambrun T, Hooks D, Frontera A, Takigawa M, Bourier F, Kitamura T, Lam A, Martin C (2018). Atrial fibrillation mechanisms and implications for catheter ablation. Front Physiol.

[24] Hertle E, Stehouwer CD, van Greevenbroek MM (2014). The complement system in human cardiometabolic disease. Mol Immunol.

[25] Dernellis J, Panaretou M (2006). Effects of C-reactive protein and the third and fourth components of complement (C3 and C4) on incidence of atrial fibrillation. Am J Cardiol.

[26] Szebeni J (2014). Complement activation-related pseudoallergy: a stress reaction in blood triggered by nanomedicines and biologicals. Mol Immunol.

[27] Lappegard KT, Garred P, Jonasson L, Espevik T, Aukrust P, Yndestad A, Mollnes TE, Hovland A (2014). A vital role for complement in heart disease. Mol Immunol.

[28] Hu YF, Chen YJ, Lin YJ, Chen SA (2015). Inflammation and the pathogenesis of atrial fibrillation. Nat Rev Cardiol.

[29] Zhang P, Shao L, Ma J (2018). Toll-like receptors 2 and 4 predict new-onset atrial fibrillation in acute myocardial infarction patients. Int Heart J.

[30] Simsek B, Altay S, Ozbilgin N, Onat A (2018). Autoimmune activation as a determinant of atrial fibrillation among Turks: a prospective evaluation. Medicine.

[31] Fabritz L, Guasch E, Antoniades C, Bardinet I, Benninger G, Betts TR, Brand E, Breithardt G, Bucklar-Suchankova G, Camm AJ (2016). Expert consensus document: defining the major health modifiers causing atrial fibrillation: a roadmap to underpin personalized prevention and treatment. Nat Rev Cardiol.

[32] Wiersma M, Meijering RAM, Qi XY, Zhang D, Liu T, Hoogstra-Berends F, Sibon OCM, Henning RH, Nattel S, Brundel B (2017). Endoplasmic reticulum stress is associated with autophagy and cardiomyocyte remodeling in experimental and human atrial fibrillation. J Am Heart Assoc.

[33] Christians ES, Benjamin IJ (2012). Proteostasis and REDOX state in the heart. American journal of physiology Heart and circulatory physiology.

[34] Jakobsson ME, Malecki J, Falnes PO (2018). Regulation of eukaryotic elongation factor 1 alpha (eEF1A) by dynamic lysine methylation. RNA Biol.

[35] Jakobsson ME, Malecki JM, Halabelian L, Nilges BS, Pinto R, Kudithipudi S, Munk S, Davydova E, Zuhairi FR, Arrowsmith CH (2018). The dual methyltransferase METTL13 targets N terminus and Lys55 of eEF1A and modulates codon-specific translation rates. Nat Commun.

[36] Freedman RB, Desmond JL, Byrne LJ, Heal JW, Howard MJ, Sanghera N, Walker KL, Wallis AK, Wells SA, Williamson RA (2017). ‘Something in the way she moves’: the functional significance of flexibility in the multiple roles of protein disulfide isomerase (PDI). Biochim Biophys Acta Proteins Proteom.

[37] Soares Moretti AI, Martins Laurindo FR (2017). Protein disulfide isomerases: redox connections in and out of the endoplasmic reticulum. Arch Biochem Biophys.

[38] Ettelaie C, Collier MEW, Featherby S, Greenman J, Maraveyas A (2018). Peptidyl-prolyl isomerase 1 (Pin1) preserves the phosphorylation state of tissue factor and prolongs its release within microvesicles. Biochim Biophys Acta Mol Cell Res.

[39] Wang JZ (2018). A novel glucose-Pin1-eNOS-NO signaling axis links diabetes mellitus with cardiovascular diseases. Int J Cardiol.

[40] Nechama M, Kwon J, Wei S, Kyi AT, Welner RS, Ben-Dov IZ, Arredouani MS, Asara JM, Chen CH, Tsai CY (2018). The IL-33-PIN1-IRAK-M axis is critical for type 2 immunity in IL-33-induced allergic airway inflammation. Nat Commun.

